# The *Dictyostelium discoideum* genome lacks significant DNA methylation and uncovers palindromic sequences as a source of false positives in bisulfite sequencing

**DOI:** 10.1093/nargab/lqad035

**Published:** 2023-04-18

**Authors:** Robert A Drewell, Tayla C Cormier, Jacob L Steenwyk, James St Denis, Javier F Tabima, Jacqueline M Dresch, Denis A Larochelle

**Affiliations:** Biology Department, Clark University, 950 Main Street, Worcester, MA 01610, USA; Biology Department, Clark University, 950 Main Street, Worcester, MA 01610, USA; Biology Department, Clark University, 950 Main Street, Worcester, MA 01610, USA; Howard Hughes Medical Institute and Department of Molecular and Cell Biology, University of California, Berkeley, Berkeley, CA 94720, USA; Biology Department, Clark University, 950 Main Street, Worcester, MA 01610, USA; Biology Department, Clark University, 950 Main Street, Worcester, MA 01610, USA; Biology Department, Clark University, 950 Main Street, Worcester, MA 01610, USA; Biology Department, Clark University, 950 Main Street, Worcester, MA 01610, USA

## Abstract

DNA methylation, the addition of a methyl (CH_3_) group to a cytosine residue, is an evolutionarily conserved epigenetic mark involved in a number of different biological functions in eukaryotes, including transcriptional regulation, chromatin structural organization, cellular differentiation and development. In the social amoeba *Dictyostelium*, previous studies have shown the existence of a DNA methyltransferase (DNMA) belonging to the DNMT2 family, but the extent and function of 5-methylcytosine in the genome are unclear. Here, we present the whole genome DNA methylation profile of *Dictyostelium discoideum* using deep coverage replicate sequencing of bisulfite-converted gDNA extracted from post-starvation cells. We find an overall very low number of sites with any detectable level of DNA methylation, occurring at significant levels in only 303–3432 cytosines out of the ∼7.5 million total cytosines in the genome depending on the replicate. Furthermore, a knockout of the DNMA enzyme leads to no overall decrease in DNA methylation. Of the identified sites, significant methylation is only detected at 11 sites in all four of the methylomes analyzed. Targeted bisulfite PCR sequencing and computational analysis demonstrate that the methylation profile does not change during development and that these 11 cytosines are most likely false positives generated by protection from bisulfite conversion due to their location in hairpin-forming palindromic DNA sequences. Our data therefore provide evidence that there is no significant DNA methylation in *Dictyostelium* before fruiting body formation and identify a reproducible experimental artifact from bisulfite sequencing.

## INTRODUCTION

**Figure 1.**
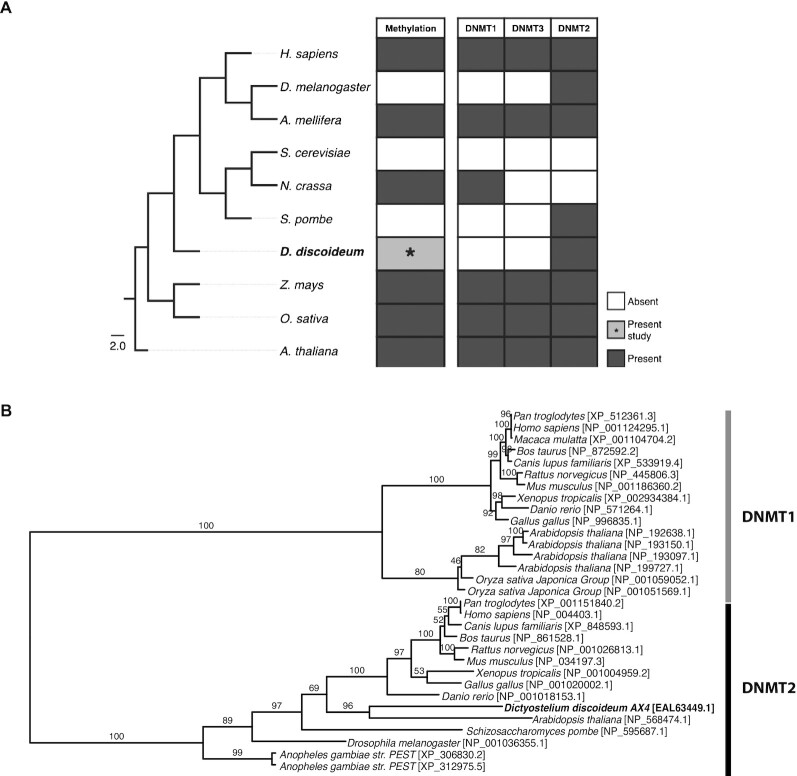
Evolution of DNA methyltransferases. (**A**) The presence (dark boxes) or absence (white boxes) of biologically significant DNA methylation and the DNA methyltransferase (DNMT) enzymes across eukaryotes. Kingdom, Animalia: *H. sapiens*, *Homo sapiens*; *D. melanogaster*, *Drosophila melanogaster*; *A. mellifera*, *Apis mellifera*. Kingdom, Fungi: *S. cerevisiae*, *Saccharomyces cerevisiae*; *N. crassa*, *Neurospora crassa*; *S. pombe*, *Schizosaccharomyces pombe*. Kingdom, Protozoa: *D. discoideum*, *Dictyostelium discoideum*. Kingdom Plantae: *Z. mays*, *Zea mays* subsp. *mays*; *O. sativa*, *Oryza sativa*; *A. thaliana*, *Arabidopsis thaliana*. The tree was generated using the NCBI taxonomy common tree (https://www.ncbi.nlm.nih.gov/Taxonomy/CommonTree/wwwcmt.cgi). **(B)** Maximum likelihood phylogenetic reconstruction of DNMT proteins including DNMA (EAL63449.1) from *D. discoideum* AX4. Bars indicate phylogenetic grouping of DNMT1 (gray) and DNMT2 (black) homologenes. Values over branches represent support percentage from 1000 bootstrap replicates. NCBI protein IDs are indicated.

DNA methylation is a post-synthetic modification that typically occurs on cytosine residues in eukaryotic plants and animals (1). Generally, DNA methylation is associated with transcriptional repression (2), but has been linked to more complex processes, including cell differentiation (3), genomic imprinting and stability (4,5), and X chromosome inactivation (6). The majority of DNA methylation is carried out by the DNA methyltransferases DNMT1, DNMT3A and DNMT3B (7). DNMT1 is responsible for maintaining a cell’s methylation profile post-replication by targeting hemi-methylated sites in the genome (8), while DNMT3A and DNMT3B methylate CpG dinucleotides *de novo*, thereby creating new epigenetic marks (9). Although DNA methylation appears to be evolutionarily conserved across a large number of eukaryotes (Figure 1), some organisms, such as *Dictyostelium discoideum* and *Drosophila melanogaster*, lack the major DNMTs while retaining the less characterized methyltransferase DNMT2 (8).

DNMT2 was originally identified based on sequence conservation of essential catalytic motifs and exhibits structural similarity to other DNMTs (10,11). It is found as a single-copy gene across the eukaryotic tree of life in protists, plants, fungi and animals (Figure 1), suggesting an important functional role (11,12). There is active debate relating to the biological function of DNMT2 and specifically the target substrates for this enzyme. To date, DNMT2 has been shown to predominantly methylate tRNAs (13), with the tRNA^Asp(GUC)^ as the preferred target substrate both *in vivo* and *in vitro* in *D. discoideum* (14). There is a growing body of evidence that in species that lack DNMT1 and DNMT3 there is no biologically significant DNA methylation (Figure 1A). Despite this observation, there is some evidence that DNMT2 may contribute to methylation of retrotransposons in the *D. melanogaster* genome (15), demonstrating a potential dual specificity for DNA and RNA substrates (16). In addition, recent studies have demonstrated that DNMT2 can efficiently methylate DNA when presented in the structural context of a tRNA (17). Currently, characterizing the presence, relevance and efficacy of DNA methylation in organisms containing only a DNMT2 methyltransferase remains an active area of research (10,11,16,18,19).

*Dictyostelium discoideum*, a eukaryotic slime mold containing a *Dnmt2* homolog (*dnmA/DDB0231095*) (20,21), is among the organisms where the presence, extent and function of DNA methylation are debated. In the initial 1991 study, *D. discoideum* was reported to lack DNA methylation, as determined by whole genome methylation-sensitive restriction enzyme analysis and high-performance liquid chromatography assays (22). Despite this early report, the confirmed existence of a DNMT2 homolog, along with *D. discoideum*’s unique AT-rich genome and an under-representation of CpG dinucleotides relative to the GpC isomer, implied the possible presence of a DNA methylation system, as methylated CpGs are inherently chemically unstable and readily mutate to TpGs (20). Accordingly, the investigation of DNA methylation in *D. discoideum* was revisited in 2006 using more advanced methodologies, including antibodies to detect 5-methylcytosine in bulk genomic DNA across distinct developmental time points (23). These studies detected overall low levels of DNA methylation in the genome and were confirmed using methylation-sensitive restriction digests targeted to retrotransposons and several other genes (23). A functional role for the DNMA enzyme in silencing of retrotransposons via the asymmetric methylation of cytosine residues was confirmed using bisulfite sequencing of specific sites (21). Furthermore, DNA methylation was demonstrated to increase through development, with the highest levels at 24 h post-starvation [although there is also evidence that this may at least in part represent cell cycle-dependent changes in DNMA regulation (14)], and knocking out *dnmA* revealed developmental defects and reduced DNA methylation (23). More recently, detailed studies examining the activity of the *D. discoideum* DNMA enzyme demonstrated that specific tRNA molecules are the preferred target substrate for this methyltransferase, but other substrates potentially remain to be characterized (14). These conflicting reports, coupled with the relatively few studies investigating DNA methylation in *D. discoideum*, reflect the uncertainty regarding the status of the DNA methylation system in this species.

To determine whether a functioning DNA methylation system is present in *D. discoideum*, we utilized whole genome bisulfite sequencing (WGBS) (24) to achieve deep coverage across multiple replicates of genomic DNA isolated from cells in an 18–24 h developmental time window. Our results show that *D. discoideum* harbors a minimal number (11 out of the ∼7.5 million cytosines in the genome) of detectable significantly methylated sites that can be reproducibly identified, many of which demonstrate low levels of methylation. Targeted bisulfite PCR (BSP) sequencing at two of these robust sites in a cluster on chromosome 4 indicates that the methylation profile does not change between vegetative and developing cells. Hairpin secondary structures generated by palindromic sequences around the 11 robust sites during the bisulfite reaction appear to be responsible for the identification of these cytosines as false mC positives. This reproducible artifact represents a potentially significant source of false positives in bisulfite sequencing, which, to our knowledge, has not previously been reported. Consequently, one must proceed with caution when identifying putative mC sites that rely on bisulfite conversion methods. In addition, a knockout of the *dnmA* gene does not result in any decrease in overall DNA methylation in the genome. Taken together, our studies provide evidence that there is no significant DNA methylation in the *D. discoideum* genome prior to fruiting body formation.

## MATERIALS AND METHODS

### Phylogenetic reconstruction of DNMTs

The sequence of DNMTA from *D. discoideum* AX4 was aligned with the homologene dataset (25) for DNMT2 from NCBI that includes 14 proteins from 10 species across the tree of life (HomoloGene: 3249) and the NCBI RefSeq ortholog dataset of DNMT1 that includes 16 sequences and 12 species (HomoloGene: 124071). Multiple sequence alignment was performed using MAFFT v7.508 (26). The maximum likelihood tree was reconstructed using IQ-Tree 2 (27) using 1000 rapid bootstrap replicates.

### Whole genome DNA sources

To investigate the potential for DNA methylation sites in *D. discoideum*, AX4 strain cells were selected for whole genome analysis. *Dictyostelium discoideum* AX4 cells have near-complete chromosome level genome assembly with genes functionally validated or predicted, making it the ideal and only current candidate for whole genome analysis. *Dictyostelium discoideum* AX4 cells were grown in 100 ml HL5 liquid cultures (Formedium Ltd, Hunstanton, UK) until near saturation. Once sufficient cell density (a minimum of 1.0 × 10^6^ cells/ml) was reached, 30 ml of the culture was transferred to 50 ml conical tubes. The cells were centrifuged for 5 min at 1000 rpm at room temperature, the medium was aspirated from the tube and the cells were resuspended in 1.5 ml of 1× Starvation Buffer (20 mM MES, pH 6.8, 0.2 mM CaCl_2_, 2 mM MgSO_4_). The resuspended cells were transferred to nitrocellulose membrane pads with 0.45 μm pores pre-wetted with Starvation Buffer, and allowed to develop over the course of 18–24 h. It should be noted that cells may have developed at different densities, which could slightly alter the timing of their development. After this time, the cells were checked to make sure there was no evidence of fruiting body formation and then scraped from the pads. gDNA was extracted with a Genesee Scientific ZR Genomic DNA Tissue MiniPrep extraction kit, utilizing the solid tissue protocol. After extraction, bisulfite sequencing was conducted to determine potential sites of methylation in the genome. As a control for bisulfite conversion, 1% lambda phage DNA (New England Biolabs) purified with PureLink Quick PCR Purification Kit (Invitrogen) was added to each gDNA sample.

### Sequencing of bisulfite-converted whole genome DNA libraries

Library construction, bisulfite conversion and sequencing were performed at the Beijing Genomics Institute. Briefly, DNA was fragmented into 100–300 bp fragments by sonication (Covaris S-2, Woburn, MA, USA). The fragmentation parameters were as follows: duty cycle, 10%; intensity, 5; cycles/burst, 200; cycles, 16; total fragmentation time, 960 s. Fragmentation was confirmed using a 2100 Bioanalyzer (Agilent Technologies, Santa Clara, CA, USA). Fragments were end repaired (Illumina) as recommended by the manufacturer. Repaired fragments were ligated with methylated sequencing adaptors using a paired end adaptor oligo kit and oligo mix 5 (Illumina). Ligated fragments were selected by gel electrophoresis and fragments of size 360 bp extracted using a QIAquick gel extraction kit (Qiagen). Size-selected fragments were exposed to a MethylMiner methylated DNA enrichment kit (Invitrogen) and then subsequently bisulfite treated using an EZ DNA methylation kit (Zymo Research, Irvine, CA, USA). Libraries were amplified using T4 polymerase (Enzymatics), and sequenced on Illumina’s HiSeq PE 150 platform.

### WGBS sequence analysis and mapping DNA methylation

Data were filtered to remove adaptor sequences, duplicate sequences, contamination and low-quality reads using BGI software. We mapped our reads onto the *D. discoideum* genome assembly 1.0 (20) using BSMAP version 2.9 (28) with seed size 12, five maximum allowed mismatches per read and a base quality cutoff of 20. The resulting SAM files were then converted to sorted BAM files using SAMtools v1.8. These files were then run through the BSMAP v2.9 methratio.py script (28), which extracts the number of converted and unconverted reads at each position and corresponding methylation ratios, sequence context, strand and coordinate for each cytosine from BSMAP mapping results. Duplicate reads were not counted and only unique mappings with at least 4× coverage were considered. Reads were similarly mapped onto the lambda phage genome (GenBank: J02459.1). From these data, we determined the number of converted and unconverted reads at each cytosine position in the *Dictyostelium* and lambda genome assemblies, accounting for the fact that each read comes from a bisulfite reaction on one strand or the other.

The average methylation level across the genome was determined by the ratio of the number of reads supporting methylation to the number of reads covering a particular cytosine site:


\begin{equation*}{{\rm Rm}}_{{\rm average}} = \frac{{{N}_{\rm m}}}{{{N}_{\rm m} + {N}_{{\rm nm}}}} \times 100\% ,\end{equation*}

where *N*_m_ represents the number of methyl-C (nonconverted) reads and *N*_nm_ represents the number of nonmethylated (converted) reads.

To identify individual cytosines that were significantly methylated in the *Dictyostelium* genome, we compared the number of converted and nonconverted reads at each site. We used only sites that had coverage of four or more reads. Methylation ratios for each cytosine in the genome were calculated along with a lower and an upper bound for the 95% confidence interval of methylation ratio using the Wilson score interval for binomial proportion (29). Only cytosines with a lower 95% confidence interval of methylation ratio bound of at least 0.05 were considered to be methylated residues. We asked how likely these counts were under a binomial test where the probability of success is one minus the conversion rate, and corrected this probability value for multiple testing (30), as previously described (31–33). From this, were able to determine the statistically significant methylated sites in the genome and the level of methylation at individual 5-methylcytosines.

### Bisulfite conversion efficiency

Bisulfite conversion efficiency was determined for each WGBS dataset from the lambda phage spike-in control, in which all cytosines should have been converted, using the following equation:


\begin{equation*}\frac{{{\mathrm{T\ count}}}}{{{\mathrm{CT\ count}}}} \times 100{\mathrm{\% }},\end{equation*}

where T count is the total number of thymine (converted cytosine) reads and CT count is the total number of cytosine (unconverted) and thymine (converted cytosine) reads at each cytosine position.

### Comparative methylome analysis

Each of the four WGBS datasets were sorted and overlapped in MATLAB using the ‘intersect’ function to isolate only robust methylated sites present in all datasets.

### DNMT knockout and homologs

AX4 cells harboring a knockout of the endogenous *dnmA* gene (23) were kindly provided by the Shaulsky lab. To investigate the presence of genes in the simultaneously sequenced AX4 wild-type (WT3) and *dnmA* knockout (KO) strains, SAMtools v1.8. was used to index the sorted BAM files of mapped reads and then extract the read depth from them, using the option *-a* to include positions at which no reads aligned. Using MATLAB, the median and mean read depths across the candidate genes were extracted and compared to the median and mean read depths across their respective chromosome to determine the presence or absence of the genes in the sequenced WT3 and KO genomes.

HMMER v3.3 (34) was used to search the *D. discoideum* proteome (GenBank: AAFI00000000.2) for candidate homologs of DNA methyltransferases using the raw HMM for the DNA methylase protein domain family (cd00315) obtained from PFAM (35).

### Bisulfite sequencing at individual robust sites

Genomic DNA was extracted from vegetative cells and cells 18–24 h into development as described earlier. These cells were distinct from those used in the whole genome analyses. Bisulfite conversion was performed on 600 ng of gDNA in each reaction using Zymo Research EZ DNA Methylation-Lightning Kit according to manufacturer’s instructions. Primers were designed using MethPrimer (36) to generate a PCR amplicon size of 406 bp around the target site at position 2524803 on chromosome 4 on the plus strand and a PCR amplicon size of 441 bp around the target site at position 2524824 on chromosome 4 on the minus strand:

Chr4 plus F: 5′-TTTATAGGTTTATAGTTGGAAATATTTTAGTAATA-3′
Chr4 plus R: 5′-CTAATATTTTACCTCATTTAAATAATTTTTT-3′
Chr4 minus F: 5′-AATAAATATATTAATATTGGTGTTTTATTTTATTTG-3′
Chr4 minus R: 5′-TACATTACTCTAATAATATTCTATAAACCTACAACT-3′

PCR products were ligated into the pGEM^®^-T Easy Vector (Promega) and sequenced as previously described (37). Sequences were analyzed using the Kismeth web-based tool (38), with settings for minimum fraction of positive matches set to 0.6 and minimum fraction of length set to 0.5.

### Secondary structure analysis of identified palindromic sequences

Secondary structure predictions were generated using default settings on the RNAstructure Fold web server (39) with nucleic acid type set to DNA. The sequence input was limited to the palindromic region including the cytosine(s) of interest.

### Search of palindromic sequences across AX4 genome

Palindromes were screened using the R package Biostrings (40). The function findPalindromes was used to search palindromes across all six main chromosome sequences of *D. discoideum* AX4. A minimum length of 19 bp and no limit for maximum length or number of loops were used. All nested palindromes were removed, leaving only the longest palindromes. The quantiles of predicted palindrome length were calculated with R Statistical Software 4.2.2 (41). Violin plots of predicted palindrome length distributions were created with ggplot2 (42). Each mC identified in at least two of the methylomes analyzed was mapped to determine whether the mC is located within a palindrome.

## RESULTS AND DISCUSSION

### Whole genome mapping, conversion and coverage rates

**Figure 2.**
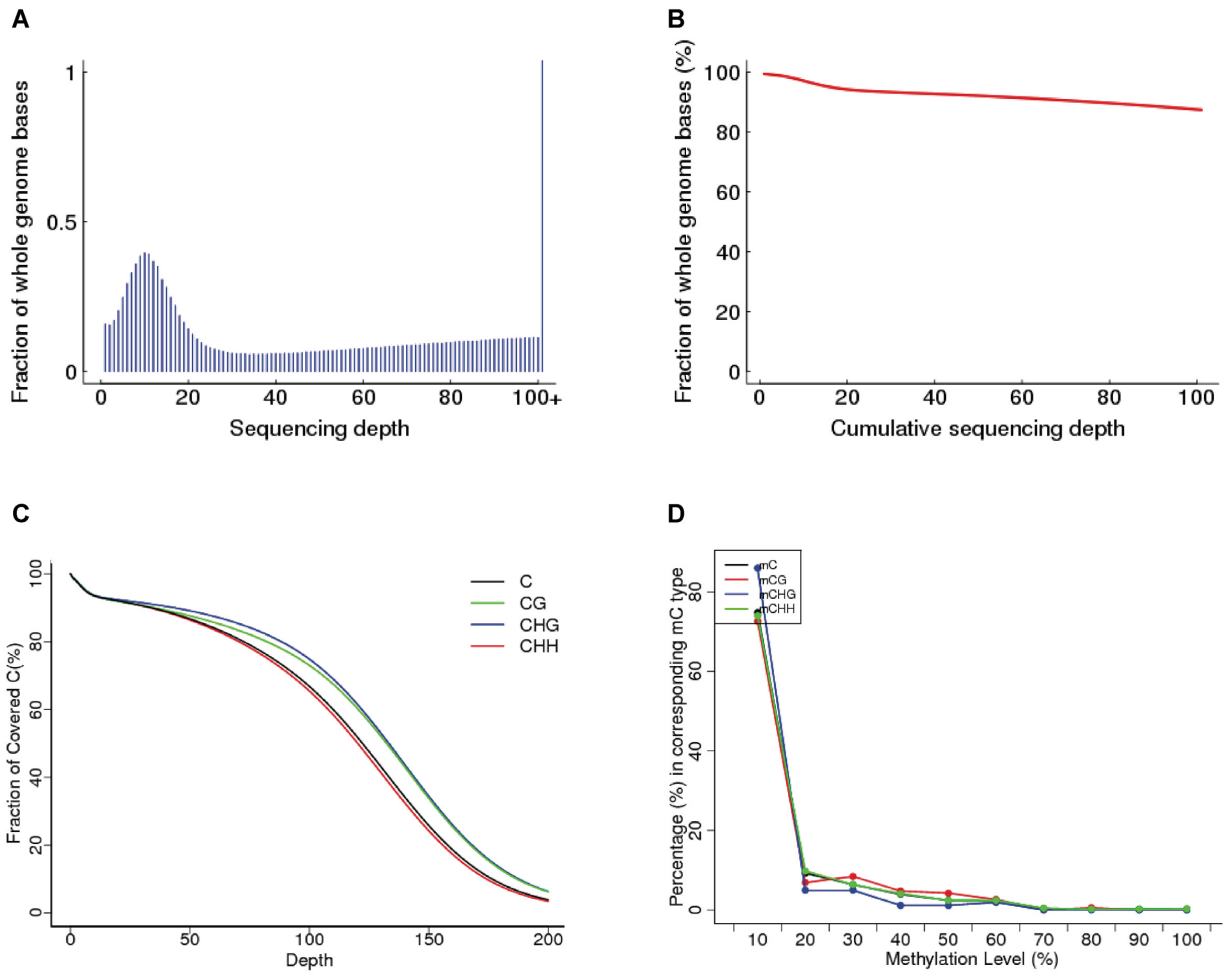
Sequencing depth and cumulative coverage of the *Dictyostelium* methylome. (**A**) Sequencing depth distribution across the entire genome. (**B**) Cumulative coverage across the entire genome, e.g. ∼90% of sites are covered by 100 or more reads. (**C**) Percentage of cytosines that have a certain level of coverage, e.g. ∼80% of sites have coverage of 75 reads. The profile for cytosines in different sequence contexts (CG, CHG and CHH, where H = non-G base) was similar. (**D**) Overall methylation level at methylcytosines. The methylation level at the individual mCs is calculated and organized in 10% bins (i.e. 0–10%, 91–100%). Only mCs covered by at least 4 and no more than 1000 reads are used in this calculation. The vast majority of mCs, irrespective of sequence context, have <10% methylation level.

Sequencing of bisulfite-converted genomic DNA from *D. discoideum* AX4 WT cells grown to between 18 and 24 h of post-starvation development, and spike-in control lambda bacteriophage DNA, across three replicate experiments generated a total of 239.2 million reads after quality control (see the ‘Materials and Methods’ section for details). This time window for the cells was chosen to ensure temporal heterogeneity of the gDNA sample. Previous studies have reported increasing levels of methylation as development progresses when samples were collected at precise time points (0, 12 and 24 h post-starvation), with the highest levels of DNA methylation reported in cells 24 h post-starvation (23). The results in our current study are not directly comparable to that study, as the gDNA samples were collected across an 18–24 h developmental time window, prior to the formation of the fruiting body. Of the sequencing reads, between 54.96% and 71.81% mapped to unique regions of the *D. discoideum* genome, which equates to an 18.13–66.15-fold average coverage across the six chromosomes in the genome, respectively, in each replicate (WT1, WT2 and WT3 in Table 1). All WT replicates shared a very similar sequencing profile, with >90% of the bases in the genome covered by 100 or more reads (Figure 2A and B). Over 80% of cytosines are covered by 75 or more reads, resulting in coverage of >98.87% of all cytosines in the replicate genomes with near-identical profiles for CG, CHG and CHH (H = non-G base) sequence contexts (Figure 2C). The bisulfite conversion rate in our experiments ranged from 99.30% to 99.53%, measured by the C-to-U deamination rate in the unmethylated lambda genome, indicating that the false-negative rate was <0.7% in each case (Table 1).

**Table 1.** Mapping, coverage and conversion rates for individual methylomes

Methylome	Quality reads (million)	Mapping rate (%)	Fold coverage	Bisulfite conversion rate (%)
WT1	73.3	54.96	18.13	99.52
WT2	125.4	71.81	66.65	99.53
WT3	40.5	66.38	30.13	99.30
KO	47.1	66.63	33.77	99.29

Fold coverage of the WGBS across the six chromosomes in *D. discoideum* is indicated for each methylome. The bisulfite conversion rate is calculated from the lambda DNA spike-in control included in each experiment.

### Overall genomic methylation profile

Initial methylome analysis indicates that a very small proportion of cytosines in the *Dictyostelium* genome are methylated. The average overall level of detectable methylation across the entire genome, calculated from the ratio of C reads to total reads (see the ‘Materials and Methods’ section for details), is <0.52% in every replicate, with a similar profile across all chromosomes and mitochondrial DNA. It should be noted that this may include a number of false-positive C reads arising from a failure to convert in the bisulfite reaction and therefore is likely an overestimate of the global methylation level. Nonetheless, methylation is detected predominantly at CHH sequences, but is also found at CG and CHG sequences (Figure 2D). Of the ∼7.5 million cytosines in the genome, only 303 (∼0.004% of all cytosines in WT2), 2135 (∼0.028% of all cytosines in WT3) and 3432 (∼0.045% of all sites in WT1) are significantly methylated (Table 2). This methylome profile mirrors previous studies that have reported global methylation levels in the *Dictyostelium* genome of 0% (22), 0.14% (23) and 0.20% (21) using different methodologies, indicating that DNA methylation is very rare in this species. These results are strikingly different from the methylation profile observed in other eukaryotes. In mammals, 60–90% of CpGs can be methylated (43), while in Hymenoptera insects 0.51–0.67% of CpGs are methylated (31,32). The sparse 0.004–0.045% of mCs detected in the three WT *Dictyostelium* genome samples collected over an 18–24 h post-starvation developmental time window is therefore lower than the level found in other eukaryotic organisms with *bona fide* DNA methylation. Among the individual sites that demonstrate a significant level of methylation in our study, the vast majority are methylated in <10% of reads, but cytosines with up to a 100% methylation level are detected (Figure 2D). It should be noted that while low levels of methylation at individual sites may be biologically significant, they may also potentially be a result of the temporal heterogeneity of the gDNA sample analyzed and/or represent false positives.

**Table 2.** Total number of statistically significant methylated sites for each WGBS dataset

Methylome	Chr1	Chr2	Chr3	Chr4	Chr5	Chr6	Total	Fold coverage
WT1	478	838	642	559	534	381	3432	18.13
WT2	40	73	77	50	37	26	303	66.65
WT3	281	567	413	382	295	197	2135	30.13
KO	332	536	422	346	307	229	2172	33.77

The number of sites is shown for each of the six chromosomes in the *Dictyostelium* genome. The total number of sites in each methylome correlates negatively with fold coverage.

### Methylation levels at individual mCs

**Figure 3.**
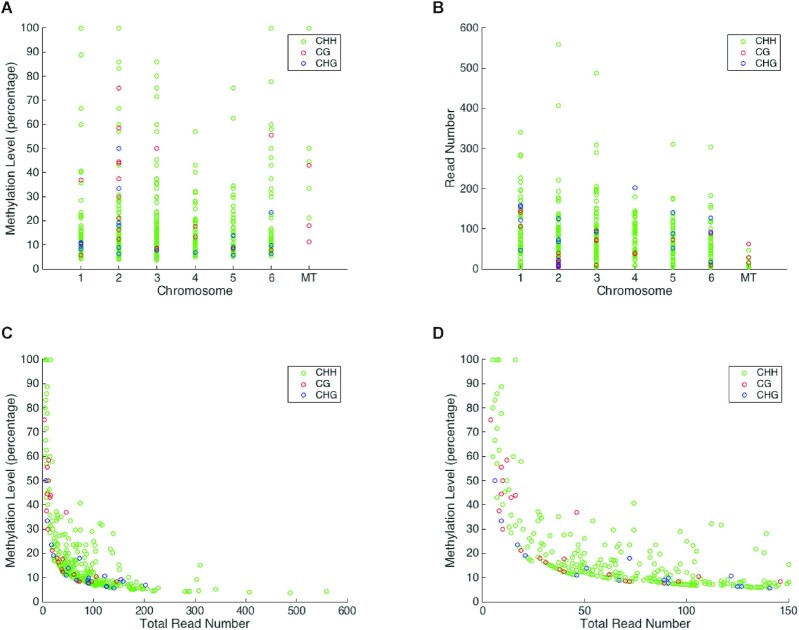
Methylation level and read depth at individual mCs. (**A**) The methylation level at the individual cytosine residues with statistically significant levels of DNA methylation is depicted for each of the six genomic chromosomes and the mitochondrial DNA. Only mCs covered by at least 4 and no more than 1000 reads are shown. (**B**) The total read number for the individual methylated sites. The vast majority of mCs, irrespective of sequence context, have <100 total reads. (**C**) The methylation level (%) plotted against the total read number for the individual methylated sites demonstrates a clear inverse relationship between read depth and methylation level at the mCs, with sites showing higher levels of methylation having lower read depth and vice versa. (**D**) This inverse relationship is particularly clear when we just consider sites with a total read depth of 150 or lower. All the methylated sites in the genome with a methylation >40% have <25 total reads.

In order to further investigate the identified cytosine residues with statistically significant levels of DNA methylation, we analyzed their profile across the genome. All chromosomes and the mitochondrial DNA show a wide range of methylation levels for mCs, with mCHH sites carrying the broadest range (Figure 3A). In parallel, we plotted the total read number for each of these mCs (Figure 3B) and compared this to methylation level (Figure 3C and D). Strikingly, there is a clear inverse relationship between read depth and methylation level at the mCs, with sites showing higher levels of methylation having lower read depth and vice versa (Figure 3C). This pattern is particularly clear if we just consider sites with a read depth below 150 total reads (Figure 3D). This potentially indicates that the high level of methylation detected at some of these mCs may simply reflect the overall low number of reads at these sites and opens up the clear possibility that the residues identified as methylated may be false positives. The clear negative correlation between the number of mCs identified in each replicate and the overall fold coverage of the genome (Table 2) suggests that a higher number of reads at each position potentially eliminate skewed methylation ratios that may otherwise appear artificially high.

### Knockout of DNMA does not alter overall methylation profile

**Figure 4.**
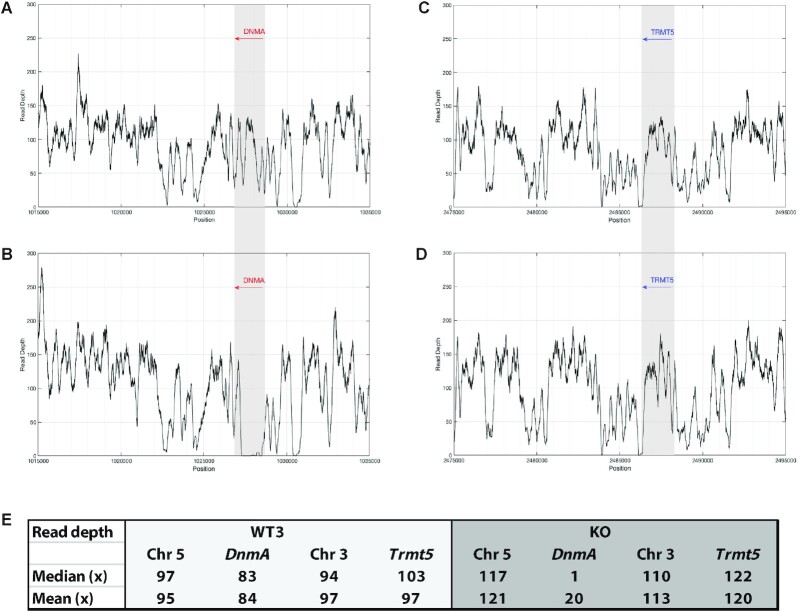
Confirmation of *dnmA* knockout. Read depths across the *dnmA* gene coordinates and the surrounding sequence in (**A**) the WT3 genome and (**B**) the KO genome. The read depth diminishes at the 5′ and 3′ ends of the *dnmA* gene in the KO, consistent with where the substituted fragment of *dnmA* begins and ends [nucleotides 80–1292 relative to the first ATG (23)]. Read depths across the *trmt5* gene coordinates and the surrounding sequence in (**C**) the WT3 genome and (**D**) KO genome demonstrate no clear difference. (**E**) The median and mean read depths across the *dnmA* and *trmt5* genes in WT3 and KO genomes. The median and mean read depths for the *trmt5* gene were in a similar range to the read depths across the entirety of chromosome 3 in both genomes, indicating that the *trmt5* gene was present, as expected, in KO and WT3. In contrast, the median and mean read depths for the *dnmA* gene were markedly lower in the KO strain, when compared to the entirety of chromosome 5. This pattern was not observed in the WT3 gene, confirming that the *dnmA* gene is deleted only in the KO strain as previously described (23).

In addition to the three replicates of the WT AX4 methylome, we also performed the same WGBS experiment on an AX4 strain harboring a knockout of the sole DNA methyltransferase (*dnmA*) in *Dictyostelium* (23). Once more, the overall sequencing (Table 1) and methylation profile (Table 2) in the KO strain was similar to the three WT methylome replicates, with a total of 2172 significant mCs identified. One potential explanation for this observation is that the knockout of *dnmA* was incomplete, in which case the DNMA enzyme may still be functionally produced in these cells. To address this possibility, we analyzed the sequencing reads in the WT3 and KO WGBS datasets by extracting the mean and median read depths across the coordinates of the *dnmA* gene (Chr. 5:1026957–1028573) and compared them to the depth across the entire chromosome 5 (Figure 4). The *dnmA* gene appeared to be knocked out as previously described (23), with a median coverage of 1× across the *dnmA* gene in the KO strain compared to a median of 117× across the entire chromosome (Figure 4B and E). In contrast, the median coverage in the WT3 genome was 83× across the *dnmA* gene and 97× across the entire chromosome, respectively, indicating that the gene is present in the WT as expected (Figure 4A and E). As a control, we performed a similar analysis at the *trmt5* gene (Chr. 3:2486411–2488090, DDBG0279739), which is annotated as a putative tRNA methyltransferase in the *Dictyostelium* genome (44). At the *trmt5* gene, no obvious difference in read depth was detected between KO and WT3 strains (Figure 4C and D). Taken as whole, these results indicate that the *dnmA* gene is deleted in the KO strain, but this has no impact on the overall methylation profile of the genome when compared to WT. This discovery suggests that DNMA is not actively involved in the methylation of DNA in the *Dictyostelium* genome and is therefore consistent with earlier studies indicating that the DNMA enzyme exhibits specificity for tRNA molecules as a preferred target substrate (14).

### Identification of 11 robust sites resistant to bisulfite conversion

The four methylome datasets were analyzed to identify only significantly methylated sites present in all of the methylomes. Despite the fact that the samples are not uniform, as they were collected across an 18–24 h developmental time widow (see the ‘Materials and Methods’ section for details), this analysis was conducted under the assumption that cytosines that consistently appear across the different methylomes are the most likely to represent true methylated cytosines. Only 11 such cytosines located on four different chromosomes were identified with a statistically significant methylation level in all datasets (Table 3). Excluding the KO dataset from this analysis resulted in the identification of only one additional site on chromosome 6 at position 2767592. Intriguingly, 7 of the 11 robust sites lie within three tight genomic clusters located on chromosomes 2 and 4 (Table 3).

**Table 3.** Statistically significant methylated cytosine positions on each chromosome in *D. discoideum* determined by bioinformatic analysis of four replicate methylomes

Chromosome	1	2	3	4	5	6	Total
Methylated sites	0	6	1	3	1	0	11
Genomic		2831383	5720793	76958	1898399		
position		3200390		2524803			
		3200403		2524824			
		8101959					
		8101986					
		8102002					

The genomic position of the 11 cytosine residues identified as methylated in all four methylomes is shown.

The 11 robust methylated sites were dispersed across the plus and minus strands on four different chromosomes. All sites, except the one on chromosome 5, were in 5′-CHH-3′ (H = A, C or T) sequence contexts and none were in 5′-CG-3′ sequences (Table 4), which was unsurprising given the overwhelmingly AT-rich sequence distribution in *D. discoideum*. Methylation ratios for each site varied from 0.138 to 0.5 across datasets, with many sites identified as methylated in ∼30% of reads (Table 4). A critical question emerging from these data is whether these 11 robust sites do in fact represent true mCs in the genome or false positives generated through a reproducible experimental artifact.

**Table 4.** Methylation ratios for the 11 robust methylated sites present in all datasets

Site	Strand	Context	GC (%)	WT1	WT2	WT3	KO
Chromosome 2							
2831383	−	CHH	11.94	0.429	0.318	0.350	0.211
3200390	−	CHH	10.45	0.286	0.156	0.300	0.500
3200403	+	CHH	11.94	0.333	0.277	0.350	0.375
8101959	+	CHH	17.41	0.500	0.184	0.248	0.261
8101986	−	CHH	14.43	0.200	0.292	0.279	0.138
8102002	−	CHH	13.43	0.278	0.313	0.273	0.233
Chromosome 3							
5720793	+	CHH	9.45	0.429	0.286	0.185	0.176
Chromosome 4							
76958	−	CHH	7.96	0.250	0.281	0.317	0.286
2524803	+	CHH	16.92	0.308	0.306	0.438	0.480
2524824	−	CHH	13.43	0.143	0.345	0.318	0.375
Chromosome 5							
1898399	+	CHG	10.45	0.333	0.148	0.222	0.200

The strand, sequence context, GC content calculated across the sequence 100 bp upstream and downstream of the site, and methylation ratios are reported.

### Bisulfite sequencing at individual robust sites in chromosome 4 cluster

To address this question in more detail, we further investigated two of the robust sites located in close proximity to each other using targeted BSP sequencing (see the ‘Materials and Methods’ section for full details). The two sites in question are found on opposite DNA strands, but are both located in a CHH sequence context and are only 21 bp apart (positions 2524803 and 2524824) on chromosome 4. Previous studies have indicated that overall DNA methylation increases through *Dictyostelium* development, with the highest levels in cells at 24 h post-starvation (23). We therefore analyzed the methylation pattern at and around these two sites using BSP sequencing in both vegetative cells and cells at 18–24 h of post-starvation development.

#### Plus strand site

**Figure 5.**
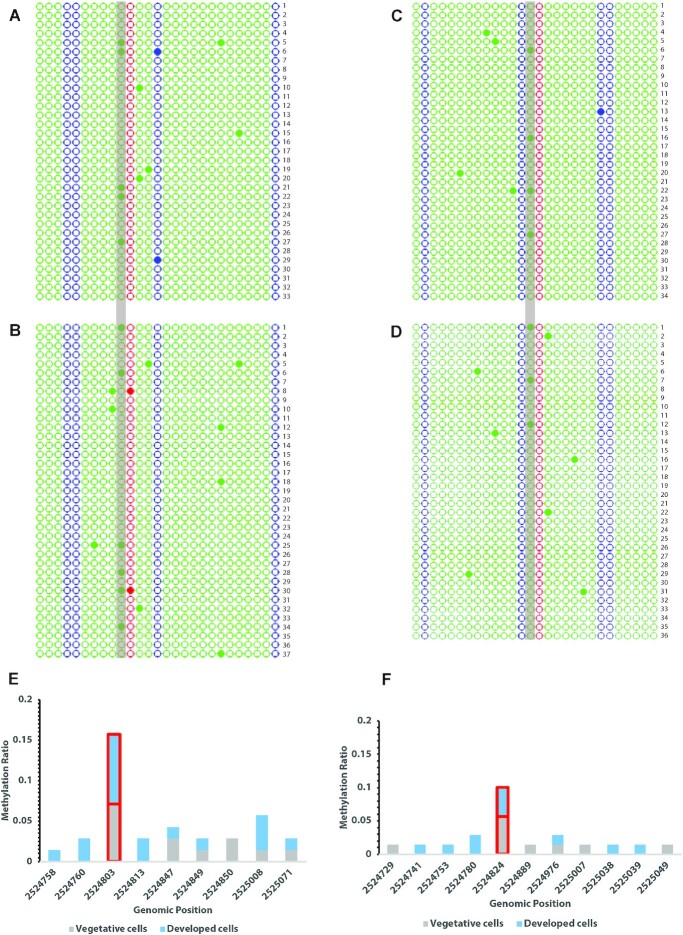
BSP sequencing at robust sites in chromosome 4 cluster. Cytosine methylation patterns of individual clones derived from sequencing are shown in the 5′–3′ orientation as single horizontal lines. Sequence context of each cytosine (shown as a circle) is indicated by color: CG (red), CHG (blue) and CHH (green), where H = non-G base. The circle is filled when the cytosine is methylated. Gray bar indicates the robust target site at position 2524803 on the plus strand in vegetative (**A**) and developed (**B**) cells and at position 2524824 on the minus strand in vegetative (**C**) and developed (**D**) cells. The methylation ratios of the cytosines with any detectable methylation in the combined vegetative (gray) and developed (blue) results are shown for the plus (**E**) and minus (**F**) strand datasets, with the robust target sites highlighted in red.

For the robust target site located at position 2524803 on the plus strand, methylation was detected at only 5 out of 33 clones in vegetative cells, representing a low methylation frequency of 15.2% (Figure 5A). Methylation was also detected at five other cytosines, out of the 27 total within the BSP amplicon, at positions 2524847, 2524849, 2524850, 2525008 and 2525071. None of these cytosines were methylated in >2 of the 33 clones (Figure 5A). A similar pattern was detected in the developed cells, with methylation of the target site in only 6 out of 37 clones, equating to a 16.2% methylation frequency (Figure 5B). Methylation was also detected at seven other cytosines within the BSP amplicon in developed cells, at positions 2524758, 2524760, 2524813, 2524847, 2524849, 2525008 and 2525071. None of these sites were methylated in >2 out of 37 clones, with the exception of the site at 2525008, which was methylated in three clones (Figure 5B). The methylation frequency of the plus strand target site is not significantly different between vegetative and developed cells as determined by a chi-square goodness-of-fit test [*χ*^2^(1, *N* = 37) = 0.03, *P* = 0.8567] and therefore indicates there is no evidence of a change in methylation through development. However, combining the data from vegetative and developed cells indicates that the overall relatively low level 15.7% methylation frequency detectable at the plus strand target site is still higher than that at any other site in the amplicon, all of which fall in the range of 1.4–5.7% (Figure 5E).

#### Minus strand site

For the robust target site located at position 2524824 on the minus strand, a very similar overall profile to the plus strand site in the cluster is observed. Although only 4 out of 34 clones were methylated at the minus strand target site in vegetative cells (Figure 5C) and 3 out of 36 in developed cells (Figure 5D), this cytosine was still distinguishably more methylated than any of the other 27 cytosines in the amplicon. Methylation was detectable at 10 other cytosines in total, including at positions 2524729, 2524889, 2524976, 2525007 and 2525049 (each methylated in just one of the clones) in vegetative cells (Figure 5C), and cytosines at positions 2524741, 2524753, 2524976, 2525038 and 2525039 (each methylated in a single clone) and the cytosine at position 2524780 (methylated in two clones) in developed cells (Figure 5D). The overall methylation frequency of the minus strand target site is not significantly different between vegetative and developed cells as determined by a chi-square goodness-of-fit test [*χ*^2^(1, *N* = 36) = 0.408, *P* = 0.52282], supporting the observation from the plus strand data that there is no change in methylation during development. However, combining the data from vegetative and developed cells indicates that the overall low-level 10.0% methylation frequency detectable at the minus strand target site is above that at any other site in the amplicon, all of which fall in the range of 1.4–2.9% (Figure 5F).

### Comparison of methylation status across all datasets

Given that the two robust target sites in the chromosome 4 cluster consistently demonstrated a reproducibly higher level of methylation than any other cytosines in their genomic vicinity, we employed an integrated analysis of both our BSP and WGBS results to further address the methylation status at and around these sites. The methylation ratios for each cytosine with any detectable methylation in the BSP amplicons and/or any of the four methylomes were tabulated for the plus strand (Table 5) and minus strand (Table 6). The methylation ratios at each site were also averaged in a one-way ANOVA, which identified at least one statistically significant difference in the mean methylation ratios between the sites. Post hoc comparisons using the Tukey HSD test determined that the methylation ratio mean at the robust target site on both strands was significantly greater than the mean at each of the other sites on their respective strands, where *P* < 0.001 for each comparison. This result indicates that, with the exception of the two robust target sites on chromosome 4, every other cytosine with detectable methylation in the BSP data is likely a false positive, potentially occurring as a result of spurious low-level conversion in the bisulfite reaction. This observation is supported by the fact that all these sites were largely found to be unmethylated in the methylome datasets (Tables 5 and 6). The exception was the cytosine at position 2524847 in the plus strand data, which had methylation frequencies ranging from 6.7% to 28.7% in the methylome datasets (Table 5). However, the high frequency of 28.7% was found in the WT1 methylome, which had the lowest coverage with only 11 total reads at that site, making it the least reliable measure of methylation ratio in the WGBS datasets.

**Table 5.** Chromosome 4 cluster plus strand methylation profile

Site position			Meth. ratio				Meth. reads				Unmeth. reads				Lower meth. ratio CI			
	WT1	WT2	WT3	KO	BSP-V	BSP-D	WT1	WT2	WT3	KO	WT1	WT2	WT3	KO	WT1	WT2	WT3	KO
2524758	0	0	0	0	0	0.027	0	0	0	0	14	75	34	32	0	0	0	0
2524760	0	0.013	0.059	0	0	0.054	0	1	2	0	14	74	32	32	0	0.002	0.016	0
**2524803**	**0.308**	**0.306**	**0.438**	**0.480**	**0.152**	**0.162**	**4**	**19**	**14**	**12**	**9**	**43**	**18**	**13**	**0.127**	**0.206**	**0.282**	**0.300**
2524813	0	0.018	0	0	0	0.054	0	1	0	0	13	55	34	25	0	0.003	0	0
2524847	0.2873	0.073	0.067	0.143	0.061	0.027	3	4	2	3	8	51	28	18	0.097	0.029	0.018	0.050
2524849	0	0.019	0	0.045	0.030	0.027	0	1	0	1	11	54	30	22	0	0.003	0	0.008
2524850	0	0	0	0.095	0.061	0	0	0	0	2	11	53	30	19	0	0	0	0.027
2525008	–	0	0	0	0.030	0.081	–	0	0	0	–	34	8	9	–	0	0	0
2525071	–	0.026	0	0	0.030	0.027	–	1	0	0	–	38	13	13	–	0.005	0	0

Replicate wild-type (WT1, WT2 and WT3) and *dnmA* KO methylome data, and BSP sequencing results for vegetative (BSP-V) and developed (BSP-D) cells in the BSP amplicon around the robust target cytosine at position 2524803 (indicated in bold) on the plus strand in the chromosome 4 cluster. The overall methylation ratio for each site is shown for each individual dataset. The methylated reads, unmethylated reads and lower bound of methylation ratio confidence interval (CI) of each site that was methylated in at least one BSP clone (shown in Figure 5A and B) are listed for the methylome datasets. Sites 2525008 and 2525071 were absent in the WT1 mapping results, likely due to poor coverage at this location.

**Table 6.** Chromosome 4 cluster minus strand methylation profile

	Meth. ratio						Meth. reads				Unmeth. reads				Lower meth. ratio CI			
Site position	WT1	WT2	WT3	KO	BSP-V	BSP-D	WT1	WT2	WT3	KO	WT1	WT2	WT3	KO	WT1	WT2	WT3	KO
2524729	0.043	0	0	0	0.029	0	1	0	0	0	22	79	31	35	0	0	0	0
2524741	0	0.012	0.061	0	0	0.028	0	1	2	0	25	80	31	36	0	0.002	0.017	0
2524753	0	0	0.031	0.027	0	0.028	0	0	1	1	28	76	31	36	0	0	0.006	0.005
2524780	0	0.081	0.036	0.108	0	0.056	0	6	1	4	29	68	27	33	0	0.038	0.006	0.043
**2524824**	**0.143**	**0.345**	**0.318**	**0.375**	**0.118**	**0.083**	**3**	**23**	**7**	**12**	**18**	**45**	**15**	**20**	**0.050**	**0.242**	**0.164**	**0.229**
2524889	0.067	0.017	0	0	0.029	0	1	1	0	0	14	58	25	28	0.012	0.003	0	0
2524976	0	0	0.059	0	0.029	0.028	0	0	1	0	5	38	16	13	0	0	0.010	0
2525007	0	0	0	0	0.029	0	0	0	0	0	6	30	7	8	0	0	0	0
2525038	0	0.059	0	0	0	0.028	0	2	0	0	6	34	9	10	0	0.016	0	0
2525039	0	0	0	0	0	0.028	0	0	0	0	6	36	9	10	0	0	0	0
2525049	0	0.029	0	0	0.029	0	0	1	0	0	6	38	9	11	0	0.005	0	0

Replicate wild-type (WT1, WT2 and WT3) and *dnmA* KO methylome data, and BSP sequencing results for vegetative (BSP-V) and developed (BSP-D) cells in the BSP amplicon around the robust target cytosine at position 2524824 (indicated in bold) on the minus strand in the chromosome 4 cluster. The overall methylation ratio for each site is shown for each individual dataset. The methylated reads, unmethylated reads and lower bound of methylation ratio confidence interval (CI) of each site that was methylated in at least one BSP clone (shown in Figure 5C and D) are listed for the methylome datasets.

For the two robust target sites, the methylation ratios were noticeably higher in the WGBS datasets than the BSP data. The plus strand cytosine at position 2524803 was identified as methylated in 30.6–48.0% of reads in the different WGBS datasets, but only in 15.2% (vegetative) and 16.2% (developed) of the BSP clones (Table 5). Similarly, the minus strand cytosine at position 2524824 was identified as methylated in 14.3–37.5% of reads in the different WGBS datasets, but only in 11.8% (vegetative) and 8.3% (developed) of the BSP clones (Table 6). These results indicate that the WGBS methylation ratios may represent an overestimate of the methylation frequency at the robust sites, potentially caused by relatively low read depth at these positions. One question we must consider is whether such low-level cytosine methylation is distinguishable from false positives.

### Palindromic sequences as a source of false positives

In order to address this question, we investigated the hypothesis that the consistent methylation of the 11 sites, and perhaps the genomic clustering observed among some of them, is the result of a molecular characteristic they all share, which confers increased resistance to bisulfite conversion. The detection of 5mC using bisulfite conversion was first shown by Frommer *et al.* (45) and Clark *et al.* (46) 30 years ago. The method relies upon the complete deamination of cytosine to uracil by modification with bisulfite, followed by PCR of the modified genomic DNA and sequencing of the products. 5mC does not react with bisulfite (47) and therefore in the final sequence analysis all of the original unmethylated cytosines appear as thymines while 5mC residues are displayed as cytosines. In contexts such as the *Dictyostelium* genome, where there are potentially very low levels of methylation and/or the methylation is variable in a given DNA sequence, it becomes difficult to determine whether the detected cytosines represent *bona fide* methylation or false-positive unconverted cytosines generated as an experimental artifact. In an attempt to identify anything unusual about the genomic regions around the methylated sequences in *Dictyostelium*, GC content was calculated in the 100 bp upstream and downstream of each of the 11 sites and was found to be markedly lower (range 7.96–17.41%) than the 23% average across the entire genome (Table 4). However, this was not particularly unexpected as all of the sites are located in non-protein-coding regions of the genome, where lower GC content is typical in the AT-rich *D. discoideum* genome.

**Figure 6.**
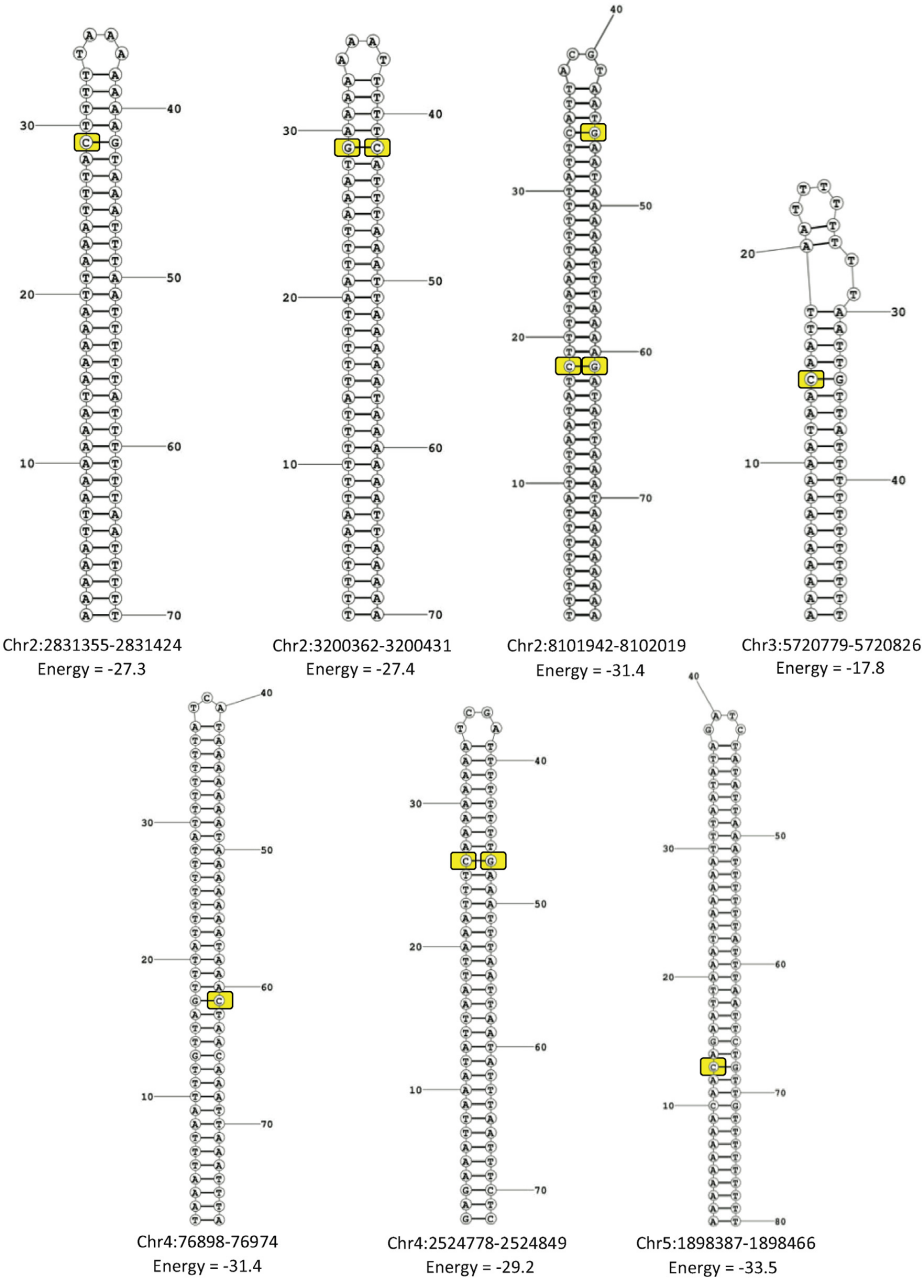
Predicted stem–loop structures of palindromic sequences around the 11 significantly methylated sites. The lowest energy conformation and its minimum free energy is shown. Nucleotides identified as significantly methylated are highlighted in yellow. For clarity, only one DNA strand from each palindromic region is shown. As a result, highlighted guanine (G) residues represent a base paired with a ‘methylated’ cytosine (C) on the complementary strand in the original dsDNA of the genome.

The most remarkable pattern observed across the 11 sites was their consistent location within palindromic sequences 48–80 nucleotides in length. The predicted secondary structures of the seven palindromic regions harboring all 11 sites are shown in Figure 6. Given these predictions, there is a strong possibility that, as single-stranded DNA (ssDNA) molecules generated in the bisulfite treatment protocol, these sequences form rigorous hairpin-like secondary structures (Figure 6). As bisulfite-mediated deamination of cytosine to uracil only occurs in ssDNA, it is possible that the stem–loop double-stranded secondary structures shield to some extent the cytosines within them from bisulfite conversion, leading to higher levels of nonconversion than other sites. Incomplete separation of strands or reannealing of strands during the bisulfite conversion reaction has been shown to produce false-positive artifacts (48,49). In the case of the 11 sites in our datasets, it is likely that the denaturation step of the bisulfite reaction is separating the plus and minus strands adequately, but the ssDNA may be rapidly reannealing to itself in these palindromic regions, essentially forming a stretch of double-stranded DNA (dsDNA) that inhibits bisulfite conversion at those sites (Figure 6).

One issue with this explanation is that in cases where there are other cytosines within the palindromic region, the other cytosines are not necessarily showing high rates of nonconversion across the four methylomes. Notably there were very few other ‘converted’ cytosines (20 in total across the combined 495 nucleotides) within the seven palindromic regions. As a result of the strongly AT-rich composition of these regions, there were only between two and eight total cytosines within any one palindromic sequence across the plus or minus strands (Figure 6). An analysis of the WGBS methylation ratios of the other ‘converted’ cytosines within the palindromic sequences actually reveals some degree of nonconversion, with these residues meeting our criteria for statistically significant methylation in zero to three of the four methylomes, with a median of two (data not shown). Strikingly, there is one characteristic shared among all the cytosines predicted to be within the loop of the hairpin structure (which account for 7 of the 20 ‘converted’ residues; Figure 6): they were all completely converted in the bisulfite reaction in every methylome and therefore identified as unmethylated. As the loop of the hairpin would essentially remain single-stranded and susceptible to bisulfite conversion at the usual rate, while cytosines within the double-stranded stem would be more resistant to conversion, this supports the idea that the secondary structure strongly impacts bisulfite conversion efficiency. We therefore conclude that the methylation detected at the 11 robust sites in the *D. discoideum* genome represents false-positive artifacts resulting from the high tendency of the regions around these sites to form localized dsDNA that inhibits bisulfite conversion.

### Majority of identified methylated cytosines are located in palindromic sequences

**Figure 7.**
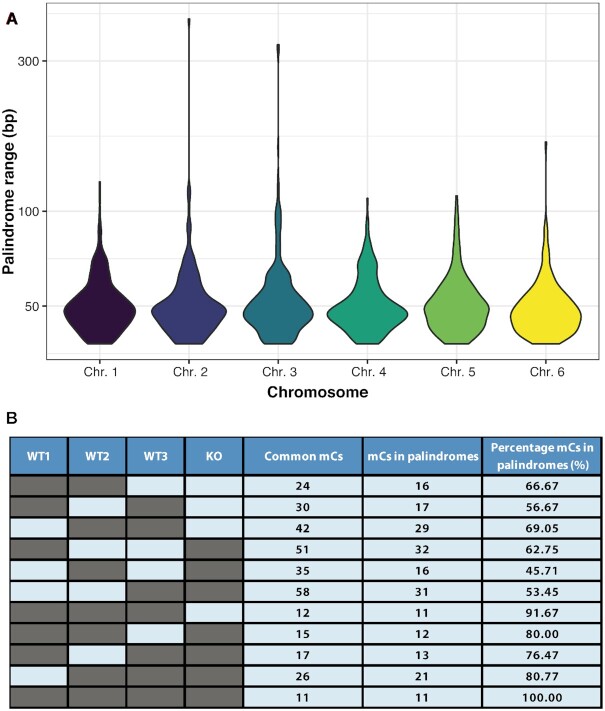
Majority of identified methylated sites are located in palindromic sequences. (**A**) A total of 1770 palindromic sequences are identified across the six *Dictyostelium* chromosomes, with a similar size profile on each chromosome. (**B**) The number of 5mCs shared between at least two of the four methylomes (WT1, WT2, WT3 and KO) is shown (common mCs) along with the number that are located in one of the 1770 identified palindromic sequences (mCs in palindromes). The percentage located in palindromic sequences increases as the stringency increases from two to three methylomes and culminates with 100% of the 11 sites found in all four methylomes being located in palindromic sequences.

To further investigate the potential for palindromic sequences to act as a source of false positives, we analyzed their prevalence across the entire *Dictyostelium* genome. We identified 1770 palindromic sequences (see the ‘Materials and Methods’ section for details) distributed across all six chromosomes (see Supplementary Table S1 for a complete list). The overall profile of the palindromic sequences in terms of size was also similar across all six chromosomes, with the vast majority ranging from 19 to 80 bp (Figure 7A). We then examined the frequency that a residue identified as a 5mC in at least two of the methylomes was found in one of these palindromic sequences (see Supplementary Table S2). Strikingly, in every possible pairwise three-way and the single four-way methylome analysis, at least 45.71% of the identified common 5mCs were located in palindromic sequences (Figure 7B). Furthermore, as the stringency of the analysis is increased, by requiring a 5mC to be shared between more methylomes, so did the percentage of 5mCs located in palindromic sequences. If just two methylomes are considered, the range is 45.71–69.05%, for three methylomes it is 76.47–91.67% and for the 11 5mCs shared in all four methylomes 100% are in palindromic sequences (Figure 7B). These results strongly support the hypothesis that palindromic sequences are a potentially significant source of false-positive artifacts in bisulfite conversion experiments.

## CONCLUSIONS

The paucity of studies investigating DNA methylation in *D. discoideum* has prohibited our detailed understanding of the evolution and function of eukaryotic DNA methylation with reference to *D. discoideum*’s unique phylogenetic position. That is, *D. discoideum* diverged soon after the plant–animal split and is therefore closely related to two kingdoms with largely present or absent DNA methylation systems (i.e. plants and fungi, respectively) (20) (Figure 1). Our results in this study indicate that there is no significant DNA methylation in *Dictyostelium* and that any experimentally detectable methylation at cytosine residues is likely a result of reproducible artifacts and/or low read depth in the whole genome analyses. Previous studies have addressed some of the potential sources of artifacts in bisulfite sequencing (49), identified critical experimental parameters (48) and offered approaches to resolve such artifacts in WGBS studies (50–52). The results we present in this work reveal an additional potentially significant source of false-positive artifacts in bisulfite sequencing, which, to our knowledge, has not previously been reported.

Our findings support the earlier observation that the genome of *D. discoideum* is largely devoid of methylation (22), but contradict the results obtained by Kuhlmann *et al.* (21) and Katoh *et al.* (23), in which they estimated 0.03–0.20% of all cytosines in the genome were methylated with a detectable increase between vegetative and developing AX4 cells (23). It should, however, be noted that the results of our current study may not be directly comparable to those in earlier studies as the gDNA samples we analyzed were collected over an 18–24 h post-starvation time window, prior to fruiting body formation. Combining our new data with the knowledge that there is a functional DNMT2 homolog (DNMA) with potentially broad substrate recognition in *D. discoideum* opens up the exciting possibility of further dissecting this evolutionarily conserved epigenetic system. It will be critical for future studies to focus on further characterization of the functional activity of the DNMA enzyme, in particular with reference to previously confirmed tRNA substrates (14), and investigation of the associated phenotypic impacts in the *dnmA* KO background in *Dictyostelium*.

## DATA AVAILABILITY

The sequencing datasets supporting the results of this article are available at the NCBI Sequence Read Archive under BioProject accession number PRJNA946158.

## Supplementary Material

lqad035_Supplemental_Files
