# Supplementary material for: The Dictyostelium discoideum genome lacks significant DNA methylation and uncovers palindromic sequences as a source of false positives in bisulfite sequencing
Source: NAR Genom Bioinform. 2023 Apr 18;5(2):lqad035. doi: 10.1093/nargab/lqad035 (PMC10111430; doi:10.1093/nargab/lqad035)
Supplement: lqad035_Supplemental_Files [file lqad035_supplemental_files.zip › Supporting Information.docx]

**Supporting Information**

**Table S1. Identified palindromic sequences in the *Dictyostelium* genome.**

The chromosome, start and end coordinates, width and sequence are listed for all 1770 identified palindromic sequences.

**Table S2. 5mC residues identified in at least two of the methylomes located in palindromic sequences.**

Each tab of the spreadsheet represents a different two-way, three-way or four-way methylome analysis. The genomic position of the 5mC (x) is shown, along with the chromosome, start position, end position, width and sequence of the palindrome in which the 5mC is located.
